# Trospium Chloride Transport by Mouse Drug Carriers of the Slc22 and Slc47 Families

**DOI:** 10.3390/ijms22010022

**Published:** 2020-12-22

**Authors:** Matthias Gorecki, Simon F. Müller, Regina Leidolf, Joachim Geyer

**Affiliations:** Institute of Pharmacology and Toxicology, Faculty of Veterinary Medicine, Justus Liebig University Giessen, 35392 Giessen, Germany; matthiasgorecki@gmail.com (M.G.); Simon.Mueller@vetmed.uni-giessen.de (S.F.M.); Regina.Leidolf@vetmed.uni-giessen.de (R.L.)

**Keywords:** trospium, transport, OCT, MATE, drug excretion, drug transport

## Abstract

Background: The muscarinic receptor antagonist trospium chloride (TCl) is used for pharmacotherapy of the overactive bladder syndrome. TCl is a hydrophilic positively charged drug. Therefore, it has low permeability through biomembranes and requires drug transporters for distribution and excretion. In humans, the organic cation transporters OCT1 and OCT2 and the multidrug and toxin extrusion MATE1 and MATE2-K carriers showed TCl transport. However, their individual role for distribution and excretion of TCl is unclear. Knockout mouse models lacking mOct1/mOct2 or mMate1 might help to clarify their role for the overall pharmacokinetics of TCl. Method: In preparation of such experiments, TCl transport was analyzed in HEK293 cells stably transfected with the mouse carriers mOct1, mOct2, mMate1, and mMate2, respectively. Results: Mouse mOct1, mOct2, and mMate1 showed significant TCl transport with Km values of 58.7, 78.5, and 29.3 µM, respectively. In contrast, mMate2 did not transport TCl but showed MPP^+^ transport with Km of 60.0 µM that was inhibited by the drugs topotecan, acyclovir, and levofloxacin. Conclusion: TCl transport behavior as well as expression pattern were quite similar for the mouse carriers mOct1, mOct2, and mMate1 compared to their human counterparts.

## 1. Introduction

Muscarinic receptor antagonists, also referred to as antimuscarinic drugs, are typically used for pharmacotherapy of the overactive bladder (OAB) syndrome, which is characterized by urinary urgency, with or without urgency urinary incontinence [1]. Licensed drugs for this indication include oxybutynin, solifenacin, fesoterodine fumarate, and trospium chloride (further referred to as trospium). Based on their physicochemical properties these compounds can be differentiated in the group of more lipophilic tertiary amines (e.g., oxybutynin and solifenacin) and the hydrophilic positively charged quaternary amine drug trospium [2]. Based on its physicochemical properties, trospium has a generally low permeability through biomembranes. As trospium is poorly metabolized in the liver, the pharmacokinetic behavior of this compound is determined mainly by the parent compound, largely involving drug transport across the plasma membrane. Its oral bioavailability is below 10% [3], and its penetration across the blood–brain barrier is highly restricted [4]. Accordingly, central nervous system (CNS) side effects, which are typical for tertiary amine antimuscarinic drugs, are less pronounced for trospium [5,6]. The active efflux of trospium at the blood–brain barrier via the ATP-binding cassette transporter P-glycoprotein (syn. MDR1, ABCB1) seems to contribute additionally to this effect [7]. In humans, trospium is largely excreted via tubular secretion into the urine and via feces, suggesting a role of drug transporters for its excretion through the liver and kidneys. In vitro studies have shown that trospium is a transport substrate of the organic cation transporters OCT1 and OCT2 [8,9], as well as of the multidrug and toxin extrusion MATE1 and MATE2-K carriers [10,11]. OCT1 (gene symbol *SLC22A1*) is primarily expressed in the basolateral membrane of enterocytes and hepatocytes, and OCT2 (gene symbol *SLC22A2*) is typically expressed in the basolateral membrane of renal proximal tubular cells [12]. In contrast, MATE2-K (gene symbol *SLC47A2*) is predominantly expressed in the apical membrane of proximal tubular cells and MATE1 (gene symbol *SLC47A1*) is additionally located in the canalicular membrane of hepatocytes [13]. In concert, these carriers enable the transcellular transport of cationic drugs via OCT-mediated uptake at the basolateral and MATE-mediated efflux at the canalicular/apical membrane in the liver (OCT1/MATE1) and kidneys (OCT2/MATE2-K and OCT2/MATE1) [14]. This cooperative transcellular drug transport was demonstrated in OCT/MATE double-transfected cell culture models [15,16]. As an example, König et al. (2011) showed significant transcellular transport of the oral antidiabetic drug metformin in OCT1/MATE1 and OCT2/MATE1 double-transfected MDCK cells, but not in respective mono-transfected cells [16]. More recently, in the same cell culture model Deutsch et al. (2019) showed significant transcellular transport of trospium in OCT1/MATE1 and OCT2/MATE1 cells that clearly exceeded that in respective single-transfected cells [11]. Based on these data, it can be suggested that OCT1/MATE1 in the liver and OCT2/MATE1 in the kidney are involved in the active excretion of trospium in OAB patients [11]. However, in vitro transport data are sometimes difficult to extrapolate to the clinical situation in patients. In order to elucidate the role of an individual drug transporter for the overall pharmacokinetics of a drug, specific transporter inhibitors can be co-applied to healthy subjects or patients and potential changes of the pharmacokinetic parameters can be analyzed. In such a setup, the OCT/MATE inhibitor ranitidine was co-applied with trospium to healthy subjects. In this study, the renal clearance of trospium was lower by ~15% in the ranitidine co-application group, most likely pointing to a drug-drug interaction at the renal tubular efflux transporters MATE1 and/or MATE2-K [3]. However, such studies have the limitation that the co-applied inhibitor may not reach all sites of carrier expression at sufficiently high concentrations for proper transport inhibition. As an alternative strategy, carrier-deficient knockout mice are often used to estimate the role of an individual drug transporter for the overall pharmacokinetics of a drug. For example, in Oct1/2^−/−^ double knockout mice, deficient for mouse Oct1 and Oct2, renal secretion of tetraethylammonium (TEA) was completely abolished [17]; in Mate1^−/−^ knockout mice, the urinary excretion of cephalexin and metformin was significantly reduced [18,19]; and in Abcb1a,b^−/−^ double knockout mice, deficient for P-glycoprotein, brain penetration of trospium was significantly increased while its hepatobiliary excretion was reduced [7]. With a similar experimental setup, trospium distribution and excretion studies would also be of interest in respective Oct and/or Mate knockout mouse models in order to clarify the role of the deleted drug transporters for the overall pharmacokinetics of trospium. In preparation of such experiments, the present study aimed to elucidate if the mouse counterparts of human OCT1, OCT2, MATE1, and MATE2-K are also transport active for trospium. Indeed, the mouse carriers mOct1, mOct2, and mMate1 transported trospium, whereas the phylogenetically more distant mMate2 did not support trospium transport but was transport active for the cationic probe drug 1-methyl-4-phenylpyridinium (MPP^+^).

## 2. Results

**Figure 1:**
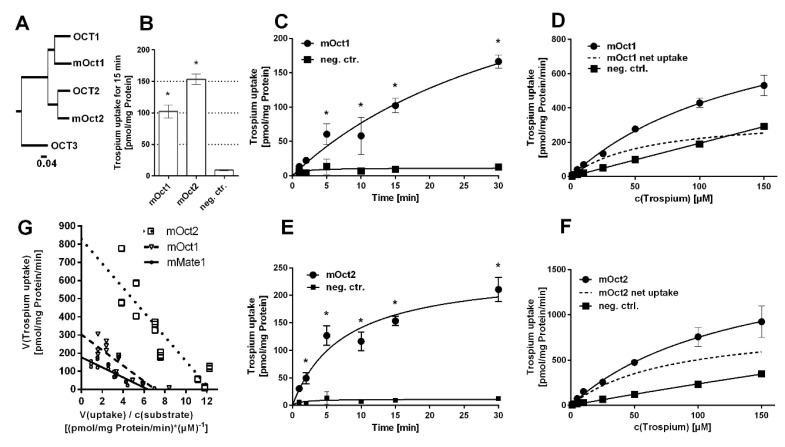
Trospium uptake by mOct1 and mOct2. (**A**) Phylogenetic tree of the human and mouse OCT/mOct carriers. The following GenBank accession numbers were used: NP_003048.1 for human OCT1, NP_003049.2 for human OCT2, NP_068812.1 for human OCT3, NP_033228.2 for mOct1, and NP_038695.1 for mOct2. Transport data represent means ± SD of representative experiments each with triplicate determinations. (**B**) Uptake of 1 µM trospium was analyzed in HEK293 cells stably transfected with mOct1 or mOct2 as indicated and in non-transfected control cells (neg. ctr.) over 15 min. * Significantly different from negative control with *p* < 0.01 (one-way ANOVA). Time-dependent uptake of 1 µM trospium over 1–30 min of substrate incubation via (**C**) mOct1 and (**E**) mOct2. * Significantly different from the respective time-point control with *p* < 0.01 (two-way ANOVA). Uptake at increasing trospium concentrations via (**D**) mOct1 and (**F**) mOct2. Non-transfected HEK293 cells served as control (neg. ctrl.). Carrier-specific uptake is indicated by dotted lines. Michaelis–Menten kinetic parameters were calculated from carrier-specific uptakes by nonlinear regression analysis. (**G**) Net uptake of trospium by mOct1 (Figure 1D), mOct2 (Figure 1F) and mMate1 (Figure 2D) were plotted as Eadie–Hofstee analysis. Intersection of regression lines with the *y*-axis indicates Vmax values; the slope indicates negative Km, and intersection with the *x*-axis indicates Vmax over Km.

**Figure 2:**
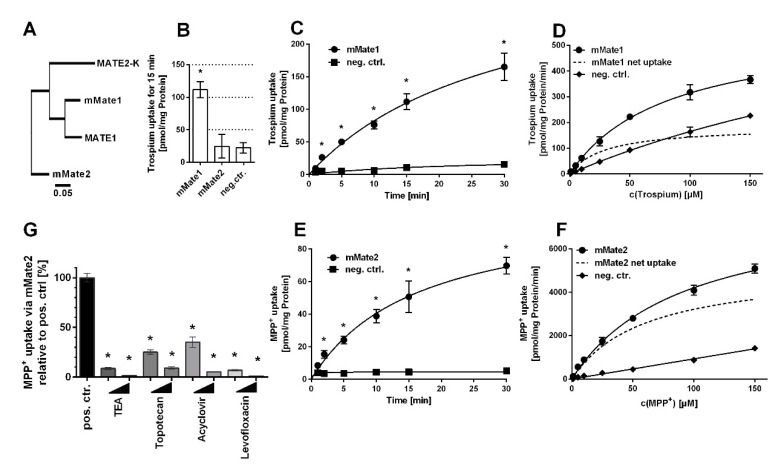
Trospium and MPP^+^ uptake by mMate1 and mMate2, respectively. (**A**) Phylogenetic tree of the human and mouse MATE/Mate carriers. The following GenBank accession numbers were used: NP_060712.2 for MATE1, NP_001093116.1 for MATE2-K, NP_080459.2 for mMate1, and NP_001028714.1 for mMate2. Transport data represent means ± SD of representative experiments each with triplicate determinations. (**B**) Uptake of 1 µM trospium was analyzed in HEK293 cells stably transfected with mMate1 or mMate2 as indicated and in non-transfected control cells (neg. ctr.) over 15 min. * Significantly different from negative control with *p* < 0.01 (one-way ANOVA). Time-dependent uptake of (**C**) trospium via mMate1 and (**E**) MPP^+^ via mMate2 over 1–30 min. * Significantly different from the respective time-point control with *p* < 0.01 (two-way ANOVA). Uptake at increasing concentrations of (**D**) trospium via mMate1 and of (**F**) MPP^+^ via mMate2 over 1 min. Non-transfected HEK293 cells served as control (neg. ctrl.). Carrier-specific uptake is indicated by dotted lines. Michaelis–Menten kinetic parameters were calculated from carrier-specific uptakes by nonlinear regression analysis. (**G**) MPP^+^ uptake inhibition via mMate2 at 10 µM and 100 µM inhibitor concentrations of TEA, topotecan, acyclovir, and levofloxacin, measured over 30 min. Cells not incubated with any inhibitor served as positive control (set to 100%). * Significantly different from positive control with *p* < 0.01 (one-way ANOVA).

The mouse carriers mOct1, mOct2, and mMate1 are orthologues to their human counterparts with high protein sequence homology (Figure 1A and Figure 2A). In contrast, mMate2 is more distant to the human MATE1 and MATE2-K carriers (Figure 2A). Of note, there seems to be no clear orthologue to human MATE2-K [20]. In order to test for trospium transport, the mouse carriers mOct1, mOct2, mMate1, and mMate2 were stably transfected into HEK293 cells and stable integration was verified by PCR expression analysis.

Then, all carriers were tested for trospium transport at 1 µM compound concentration over 15 min. Whereas mOct1, mOct2 (Figure 1B), and mMate1 (Figure 2B) showed significant (*p* < 0.01) trospium transport compared with control, mMate2 was transport negative for trospium (*p* > 0.01) (Figure 2B). Next, time-dependent trospium uptake was analyzed for mOct1 (Figure 1C), mOct2 (Figure 1E), and mMate1 (Figure 2C), and showed linear uptake for all carriers over 5 min. After 30 min of transport, the trospium accumulation rates were quite similar for mOct1, mOct2, and Mate1, all being in the range of 150–200 pmol/mg protein. Michaelis–Menten parameters were determined by measuring the trospium transport over 1 min at increasing compound concentrations, ranging from 1 µM up to 150 µM for mOct1 (Figure 1D), mOct2 (Figure 1F), and mMate1 (Figure 2D). Carrier-specific uptake was calculated by subtracting uptake into untransfected HEK293 cells (indicated by dotted lines). The following transport kinetic parameters were determined: Km of 58.7 ± 15.5 µM and Vmax of 352.9 ± 39.4 pmol/mg protein/min for mOct1, Km of 78.5 ± 25.9 µM and Vmax of 899.3 ± 139.7 pmol/mg protein/min for mOct2, and Km of 29.3 ± 6.7 µM and Vmax of 184.7 ± 14.0 pmol/mg protein/min for mMate1 (Figure 1G, Table 1). Whereas the Km values were all at comparable levels for all three carriers, mOct2 revealed by far the highest Vmax value (Figure 1F).

As mMate2 did not show any transport of trospium in repeated experiments, MPP^+^ was used as potential substrate. MPP^+^ represents a prototypic substrate for organic cation transporters. As indicated in Figure 2E, mMate2 showed significant time-dependent transport of MPP^+^ with a linear phase between 1–5 min. Transport kinetics were then measured at increasing MPP^+^ concentrations ranging from 1 µM up to 150 µM over 1 min and revealed the following Michaelis–Menten parameters: Km of 60.0 ± 5.6 µM and Vmax of 5136.0 ± 202.5 pmol/mg protein/min (Figure 2F). In order to analyze if mMate2 still represents a potential drug carrier, MPP^+^ uptake via mMate2 was inhibited by several drugs at 10 µM and 100 µM inhibitor concentrations. As shown in Figure 2G, mMate2 was significantly inhibited by tetraethylammonium (TEA), topotecan, acyclovir, and levofloxacin.

**Figure 3:**
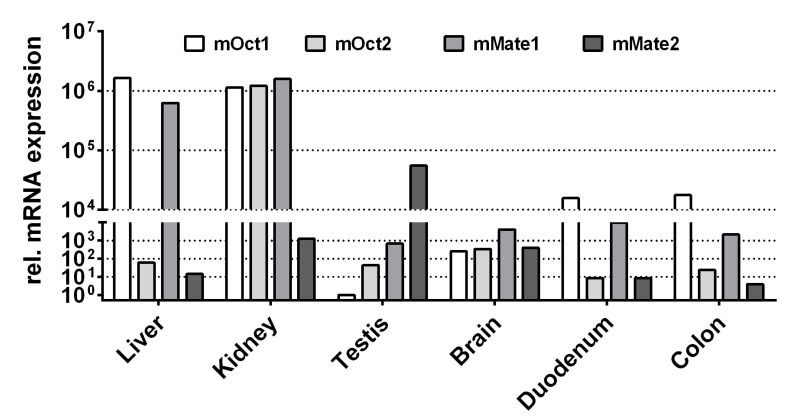
mRNA expression pattern of mOct1, mOct2, mMate1, and mMate2 in liver, kidney, testis, brain, duodenum, and colon of the mouse. The mRNA expression was analyzed by quantitative real-time PCR analysis using cDNAs from the indicated tissues of a male C57BL/6N mouse. Relative carrier expression was calculated by the 2^−ΔΔCT^ method and represents carrier expression that is *x* times higher compared with the overall lowest expression (i.e., mOct1 testis, set as calibrator). Values represent means of duplicate determinations.

Finally, the expression patterns of mOct1, mOct2, mMate1, and mMate2 were analyzed in liver, kidney, testis, brain, duodenum, and colon of a male C57BL/6N mouse. As indicated in Figure 3, mOct1 and mMate1 showed the highest mRNA expression levels in liver and kidney, whereas mOct2 was predominantly expressed only in the kidney. In contrast, mMate2 was highest expressed in the testis and showed only marginal expression in liver and kidney.

## 3. Discussion

The pharmacokinetics of the OAB drug trospium chloride is characterized by poor intestinal absorption, predominant elimination via the urine, and additional excretion via feces depending on the route of application [21]. Recent pharmacokinetic studies in human subjects receiving 2 mg trospium via intravenous infusion showed that 65% of the dose were eliminated into the urine and only about 3% via feces within 5 days [3]. In contrast, after oral administration of 30 mg immediate release tablets, the elimination via feces (25% of the oral dose) exceeded that of the urine excretion (6% of the oral dose). From the gut, trospium is absorbed from two distinct absorption “windows” located in the jejunum and the cecum/ascending colon [22]. However, the role of drug transporters for this process is not yet finally clear [3]. From the absorbed fraction, most of the trospium drug (~80%) appears unchanged in the urine. Thereby, elimination via the kidneys largely involves tubular secretion, as indicated by the 4-fold higher renal trospium clearance compared to the average glomerular filtration rate [21]. As a cationic drug, trospium has a generally low permeability through biomembranes, and therefore, its tubular secretion depends on membrane drug transporters. As a transcellular transport of trospium was already shown in a cell culture model expressing OCT2 and MATE1, these carriers, which are expressed at the basolateral and apical membrane of proximal tubular cells [12], respectively, are likely involved in the tubular secretion of trospium [11]. In addition, MATE2-K can be supposed to be involved in this process. MATE2-K is, like MATE1, expressed at the apical membrane of proximal tubular cells [13] and is also active in transporting trospium [10]. This assumption is supported by pharmacokinetic studies in healthy subject, which showed reduced renal clearance of trospium at co-administration with the OCT/MATE inhibitor ranitidine [3]. In the liver, OCT1 and MATE1 are co-expressed at the basolateral and canalicular membranes of hepatocytes, respectively [12,13]. Both carriers can also transport trospium when expressed in cell culture models [10] and, therefore, are supposed to be involved in the hepatobiliary excretion of this drug. In addition, based on excretion data from Abcb1a,b^−/−^ knockout mice, P-glycoprotein seems to be involved in the canalicular excretion of trospium in hepatocytes [7].

In general, expression sites of mOct1, mOct2, and mMate1 reflect quite well the expression pattern of their human counterparts. Similar to human OCT2, mOct2 is predominantly expressed in the kidney [17]. However, mOct1 has a broader expression pattern and is expressed in liver (as human OCT1) and in addition in kidney and small intestine [23]. Therefore, Oct1/2^−/−^ double knockout mice are used to estimate the role of Oct-mediated drug transport for the tubular secretion of drugs [17]. More complicated is the situation for the Slc47 carriers mMate1 and mMate2. Whereas mMate1 is an orthologue to human MATE1 with a similar expression pattern in the luminal membranes of renal tubular cells and bile canaliculi [24], there seems to be no mouse orthologue for human MATE2-K [20]. In contrast, mMate2 is more distant from both, MATE1 and MATE2-K, and showed predominant expression in Leydig cells of the testis [25]. However, as mMate1 is highly expressed in liver and kidney, Mate1^−/−^ knockout mice are considered as an appropriate model to study the pharmacokinetic role of MATE1 and MATE2-K in vivo [18,26].

Based on the data from the present study, trospium transport data for the mouse carriers mOct1, mOct2, and mMate1 are comparable to the trospium transport data of their human counterparts. The first hints for transport of trospium via OCTs came from inhibition studies, where trospium inhibited the MPP^+^ transport via human OCT1, OCT2, and OCT3 with IC_50_ values of 6.2, 0.67, and 871 µM, respectively [27]. This was basically confirmed by a later study showing IC_50_ values of 18.1, 1.36, and 710 µM for OCT1, OCT2, and OCT3, respectively, in a similar experimental setup [8]. In this study, transport of radiolabeled [^3^H]trospium was additionally investigated and revealed K_m_ values of 17 and 8 µM for OCT1 and OCT2, respectively, with nearly identical V_max_ values of about 90 pmol/mg protein/min. In contrast, trospium transport via OCT3 was much lower, and so transport kinetics could not be determined [8]. Therefore, in the present study, trospium transport was analyzed only for mOct1 and mOct2, but not for mOct3. Even later, Bexten analyzed transport of trospium via different drug transporters but only could show trospium transport via OCT1 with K_m_ of 106 µM and V_max_ of 269 pmol/mg protein/min, but not for OCT2 [9]. In a study also including the MATE transporters, Chen et al. (2017) found IC_50_ values of 15.4, 7.3, 11.5, 11.6, and 5.1 µM for trospium at the carriers OCT1, OCT2, OCT3, MATE1, and MATE2-K, respectively [10]. In addition, direct transport kinetics were determined for all these carriers and revealed K_m_ values of 15.1, 0.6, 4.4, 15.4, and 8.2 µM for OCT1, OCT2, OCT3, MATE1, and MATE2-K, respectively [10]. Finally, transcellular transport measurements with trospium in OCT1/MATE1 and OCT2/MATE1 double-transfected cells confirmed trospium transport via OCT1, OCT2, and MATE1 [11]. Of note, transport kinetic data for the human carriers partly varied by a factor of 10, most likely due to different experimental conditions, regarding carrier transfection (transient or stable), time-point of analysis (ranging from 1–5 min), and analytical method ([^3^H]trospium or LC-MS/MS). Against this background, it is difficult to compare directly the transport kinetics between the human and the mouse carriers. However, in direct comparison to the study by Wenge et al. [8] with comparable experimental conditions as in the present study, the Km and Vmax values were generally higher for the mouse carriers compared to their human counterparts (Table 1).

As the absolute and relative importance of a particular drug transporter can only roughly be estimated from in vitro transport data, respective mOct1/2^−/−^, mMate1^−/−^, or combined mOct1/2^−/−^/mMate^−/−^ knockout mouse models might help to elucidate the role of these drug carriers for the overall pharmacokinetics of trospium. Based on the transport data obtained in the present study and regarding the expression of mOct1, mOct2, and mMate1, these carriers are most likely involved in enteral drug absorption, hepatobiliary excretion and tubular secretion of trospium as it was also suggested for the respective human OCT and MATE carriers. Therefore, in mOct/mMate knockout mouse models, it would be particularly interesting to analyze enteral drug absorption, hepatobiliary, and tubular secretion in comparison to wild-type mice.

In contrast to mMate1, mMate2 did not transport trospium in mMate2 stably transfected HEK293 cells. Therefore, mMate2 with its highest expression in the testis does not contribute to the distribution and excretion of trospium in the mouse. However, in the present study, MPP^+^ transport via mMate2 was demonstrated, indicating that it still represents a drug transporter. This transport was significantly inhibited by TEA, topotecan, acyclovir, and levofloxacin. This is in full agreement with a previous study that showed TEA transport via mMate2 that was significantly inhibited by cimetidine, quinidine, verapamil, and some other drugs [25].

In conclusion, the present study demonstrates for the first time transport of trospium via the mouse drug transporters mOct1, mOct2, and mMate1. Based on this data, pharmacokinetic studies with this drug can be suggested in respective mOct/mMate knockout mice in order to elucidate the role of these carriers for absorption, distribution and elimination of trospium in the mouse. Due to the similarities in the expression pattern and trospium transport behavior between the human and mouse carriers, these data then can be used for extrapolation to the situation in human patients.

## 4. Materials and Methods

### 4.1. Materials and Chemicals

All chemicals, unless otherwise stated, were obtained from Sigma-Aldrich (Taufkirchen, Germany). [^3^H]MPP^+^ (80 Ci/mmol) was obtained from PerkinElmer (Waltham, MA, USA) and [^3^H]trospium trifluoroacetate (24.6 Ci/mmol) was obtained from RC Tritec AG (Teufen, Switzerland). Unlabeled trospium chloride was kindly provided by Dr. Pfleger Arzneimittel GmbH (Bamberg, Germany). For the transport experiments, [^3^H]trospium trifluoroacetate was mixed with an excess of unlabeled trospium chloride. Due to the high excess of chloride in relation to trifluoroacetate in this preparation [^3^H]trospium chloride is regarded as the active compound. The mixture of [^3^H]trospium and unlabeled trospium chloride used for all transport measurements is referred to as trospium in the manuscript.

### 4.2. Cloning of mOct1, mOct2, mMate1, and mMate2

The full open reading frames of the mouse carriers mOct1 and mOct2 were cloned from mouse liver cDNA by RT-PCR as reported before [28]. Gene-specific forward and reverse primers were deduced from the following GenBank reference sequences: NM_009202.5 for mOct1 (*Slc22a1*) and NM_013667.3 for mOct2 (*Slc22a2*). The following gene-specific primers were used: 5′-ATT TCA AGC CAC CGC AGT TC-3′ forward and 5′-CTC CCT CTT CTC TCC ACT CT-3′ reverse for mOct1, as well as 5′-CAG CAT TTG CAA CCC TGT AG-3′ forward and 5′-GTT GGG TTG TGT GGC TTT CG-3′ reverse for mOct2. The full open reading frames of mMate1 (*Slc47a1*) and mMate2 (*Slc47a2*) were synthesized based on the reference sequences NM_026183.5 (mMate1) and NM_001033542.2 (mMate2) by BioCat GmbH (Heidelberg, Germany). All open reading frames were subcloned into the pcDNA5/FRT/TO-TOPO expression vector (Thermo Fisher Scientific, Darmstadt, Germany) under control of a CMV promoter and were sequence verified by DNA sequencing.

### 4.3. Generation of Stably Transfected HEK293 Cell Lines

For the generation of stably transfected cell lines, the Flp-In T-REx 293 host cell line (Thermo Fisher Scientific) was used as reported before [28]. Flp-In T-REx 293 cells contain a single, stably integrated Flp recombinase target (FRT) site at a transcriptionally active genomic locus that ensures high level gene expression from a target-integrated Flp-In expression vector. The expression vector pcDNA5/FRT/TO carries an FRT site and the hygromycin resistance gene. In the generated vectors, the cloned carrier cDNAs are under control of the cytomegalovirus (CMV) promoter and the tetracycline operator sequences (*tetO*_2_). In order to establish stably transfected cell lines, the carrier pcDNA5 constructs were cotransfected with the Flp recombinase expression vector pOG44 into the Flp-In T-REx 293 host cells by Lipofectamine transfection reagent according to the manufacturer’s protocol (Invitrogen, Carlsbad, CA, USA). Stable clones containing the carrier open reading frame sequences under control of the CMV/*tetO_2_* hybrid promoter were selected by culturing in selective media containing 150 µg/mL hygromycin and 50 µg/mL blasticidin. All clones were verified by quantitative mRNA expression analysis for the respective transfected carrier (see below). The stably transfected mOct1-, mOct2-, mMate1-, and mMate2-HEK293 cells were maintained in Gibco D-MEM/F12 medium (Thermo Fisher Scientific) supplemented with 10% fetal calf serum, L-glutamine (4 mM), penicillin (100 U/mL), and streptomycin (100 µg/mL) (further referred to as standard medium) at 37 °C, 5% CO_2_, and 95% humidity.

### 4.4. Transport Measurements with MPP^+^ and Trospium

For transport studies, 12- or 24-well plates were coated with poly-D-lysine for better attachment of the cells, and 4.5 or 2.25 × 10^5^ cells were plated per well, respectively. Cells were grown under standard medium, and carrier expression was induced by preincubation with tetracycline (1 µg/mL) for 72 h prior to transport experiments starting. Then, cells were washed three times with phosphate buffered saline (PBS; 137 mM NaCl, 2.7 mM KCl, 1.5 mM KH_2_PO_4_, 7.3 mM Na_2_HPO_4_, pH 7.4, 37 °C) and preincubated with sodium transport buffer containing 142.9 mM NaCl, 4.7 mM KCl, 1.2 mM MgSO_4_, 1.2 mM KH_2_PO_4_, 1.8 mM CaCl_2_, and 20 mM HEPES, adjusted to pH 7.4 for mOct and pH 8 for mMate measurements. Uptake experiments were initiated by replacing the preincubation buffer by transport buffer containing the radiolabeled test compound ([^3^H]MPP^+^ or [^3^H]trospium) and were performed at 37 °C at different time-points and substrate concentrations. For mMate2 inhibition studies, the mMate2-HEK293 cells were preincubated with transport buffer containing the inhibitor compound at 37 °C for 3 min. Then, transport measurements were started by adding the radiolabeled substrate at 37 °C over 30 min. Transport and inhibition assays were terminated by removing the transport buffer and washing five-times with ice-cold PBS. Cell monolayers were lysed in 1 N NaOH with 0.1% SDS and the cell-associated radioactivity was determined on liquid scintillation analyzer Tri-Carb 2910 TR (PerkinElmer). The protein content of the lysed cells was determined as reported before with bovine serum albumin as standard [28].

### 4.5. RNA Preparation and Quantitative Real-Time PCR Expression Analysis

Tissue samples were obtained from a male C57BL/6N mouse without any treatment. Housing was performed in a specific pathogen-free animal facility with a temperature-controlled environment and 12-h light/dark cycle, and standard laboratory food and water were provided ad libitum. The mouse was sacrificed with the sole purpose of using organs for scientific purposes (according to §4 para. 3 of the TierSchG) after consulting the Animal Welfare Officer (TSchB) of the Justus Liebig University Giessen (reference number: 639_M). Organs were conserved in RNAlater solution (Sigma) at −20 °C before total RNA was extracted using RNA tissue kit and Maxwell RSC (Promega, Walldorf, Germany). The RNA amount was determined using NanoDrop One (Thermo Fisher Scientific). The cDNA was synthesized using SuperScript III (Invitrogen) according to the manufacturer’s instructions. For quantitative real-time PCR amplification, the following TaqMan Gene Expression Assays were used: Mm00456303_m1 for mOct1, Mm00457295_m1 for mOct2, Mm00840361_m1 for mMate1, and Mm02601013_m1 for mMate2 (Thermo Fisher Scientific). Amplification of mouse beta-actin (assay number Mm00607939_s1) was used as endogenous control. For each specimen, duplicate determinations were performed in a 96-well optical plate using 5 µl cDNA, 1.25 µl TaqMan Gene Expression Assay, 12.5 µl TaqMan Gene Expression Master Mix (Thermo Fisher Scientific), and ddH_2_O to a final volume of 25 µl. Quantitative real-time PCR was performed on an Applied Biosystems 7300 thermal cycler. The plates were heated for 10 min at 95 °C, and subsequently, 40 cycles of 15 s at 95 °C and 60 s at 60 °C were applied. Relative carrier expression (ΔC_T_) was calculated by subtracting the mean signal threshold cycle (C_T_) of mouse beta-actin from the mean C_T_ value of the respective carrier. Subsequently, for each tissue, ΔΔC_T_ values were calculated by subtracting testis mOct1 ΔC_T_ (set as calibrator) from the ΔC_T_ of each individual tissue. After 2^−ΔΔCT^ transformation, data show x-fold higher carrier expression in the respective tissue.

### 4.6. Graphical and Statistical Analysis of Data

All graphs were created, and the respective statistical analysis was done by GraphPad Prism 6 software (GraphPad Software, La Jolla, CA, USA). Error bars within graphs represent means ± standard deviations (SD) of triplicate determinations. For single time-point uptake and uptake-inhibition experiments, one-way ANOVA with Dunnett’s multiple comparison post-hoc test (one-way ANOVA) compared to respective controls was calculated and significant uptake or inhibition was indicated by * representing *p* < 0.01. For time-dependent uptake experiments, every time-point was compared to its individual time-point control by two-way ANOVA and significant uptake indicated by * represents *p* < 0.01. For calculation of K_m_ and V_max_ values, Michaelis–Menten equation of GraphPad Prism 6 was applied with the following parameters: least squares (ordinary) fit, no constraints, confidence interval of parameters 95%. For Eadie–Hofstee analysis, data of the concentration-dependent net uptake of trospium by mOct1 (Figure 1D), mOct2 (Figure 1F), and mMate1 (Figure 2D) were plotted together in GraphPad Prism 6. Individual [V]data (*y*-axis) were plotted against the mean of [V] data over respective substrate concentration [S] (V/S), and a linear regression line was fitted for each data set.

## Figures and Tables

**Table 1:** Trospium transport kinetics in man and mouse.

Human Carrier	Km (µM)	Vmax (pmol/mg protein/min)	Mouse Carrier	Km (µM)	Vmax (pmol/mg protein/min)
OCT1	17 ± 5 [8]	93 ± 26 [8]	mOct1	58.7 ± 15.5	352.9 ± 39.4
	106 ± 16 [9]	269 ± 18 [9]			
	15 ± 3 [10]	1142 ± 157 [10]			
OCT2	8 ± 1 [8]	92 ± 11 [8]	mOct2	78.5 ± 25.9	899.3 ± 139.7
	0.6 ± 0.1 [10]	98 ± 22 [10]			
MATE1	15 ± 2 [10]	1083 ± 143 [10]	mMate1	29.3 ± 6.7	184.7 ± 14.0
MATE2-K	8 ± 2 [10]	297 ± 6 [10]	mMate2	No transport	No transport

## Abbreviations

OCT	Organic cation transporter
MATE	Multidrug and toxin extrusion
SLC	Solute carrier
ABCB1	ATP-binding cassette transporter B1
